# Label-Free Density Measurements of Radial Peripapillary Capillaries in the Human Retina

**DOI:** 10.1371/journal.pone.0135151

**Published:** 2015-08-07

**Authors:** Paula K. Yu, Chandrakumar Balaratnasingam, Jing Xu, William H. Morgan, Zaid Mammo, Sherry Han, Paul Mackenzie, Andrew Merkur, Andrew Kirker, David Albiani, Marinko V. Sarunic, Dao-Yi Yu

**Affiliations:** 1 Centre for Ophthalmology and Visual Science, The University of Western Australia, Australia, Perth, Australia; 2 Lions Eye Institute, The University of Western Australia, Perth, Australia; 3 School of Engineering Science, Simon Fraser University, Burnaby, British Columbia, Canada; 4 Department of Ophthalmology and Visual Sciences, University of British Columbia, Vancouver, Canada; Univ Rochester Medical Ctr, UNITED STATES

## Abstract

Radial peripapillary capillaries (RPCs) comprise a unique network of capillary beds within the retinal nerve fibre layer (RNFL) and play a critical role in satisfying the nutritional requirements of retinal ganglion cell (RGC) axons. Understanding the topographical and morphological characteristics of these networks through *in vivo* techniques may improve our understanding about the role of RPCs in RGC axonal health and disease. This study utilizes a novel, non-invasive and label-free optical imaging technique, speckle variance optical coherence tomography (svOCT), for quantitatively studying RPC networks in the human retina. Six different retinal eccentricities from 16 healthy eyes were imaged using svOCT. The same eccentricities were histologically imaged in 9 healthy donor eyes with a confocal scanning laser microscope. Donor eyes were subject to perfusion-based labeling techniques prior to retinal dissection, flat mounting and visualization with the microscope. Capillary density and diameter measurements from each eccentricity in svOCT and histological images were compared. Data from svOCT images were also analysed to determine if there was a correlation between RNFL thickness and RPC density. The results are as follows: (1) The morphological characteristics of RPC networks on svOCT images are comparable to histological images; (2) With the exception of the nasal peripapillary region, there were no significant differences in RPC density measurements between svOCT and histological images; (3) Capillary diameter measurements were significantly greater in svOCT images compared to histology; (4) There is a positive correlation between RPC density and RNFL thickness. The findings in this study suggest that svOCT is a reliable modality for analyzing RPC networks in the human retina. It may therefore be a valuable tool for aiding our understanding about vasculogenic mechanisms that are involved in RGC axonopathies. Further work is required to explore the reason for some of the quantitative differences between svOCT and histology.

## Introduction

Retinal ganglion cell (RGC) axons have immense metabolic demands and are dependent upon regional vasculature for the delivery and exchange of energy substrates [1,2]. Radial peripapillary capillaries (RPCs) form a unique plexus of capillary beds within the inner aspect of the retinal nerve fibre layer (RNFL) and are a critical source of nutrition for human RGC axons [3,4]. RPCs are confined to the posterior pole of the globe, within eccentricities surrounding the optic disk, and are morphologically distinct relative to other retinal capillary beds [5]. Using human donor eyes, our previous study provided a quantitative and topographic analysis of the RPC networks surrounding the normal human optic disk [6]. We were able to identify important correlations between RPC network morphology and RNFL thickness suggesting that neurovascular co-patterning and functional crosstalk mechanisms are critically linked to retinal homeostasis. Understanding RPC network microanatomy and delineating its pattern of perturbation following various ocular insults is therefore likely to improve our understanding about pathogenic mechanisms that are involved in RGC axonal diseases. In this study, we seek to build on this work by quantifying the spatial geometry of RPCs in the living eye.

Cause-consequence relationships in glaucoma and other diseases involving the RGC axon remain complex and unclarified [7–10]. Currently there are a number of valuable investigative tools that are employed to assess RNFL structure and macro-anatomy in the clinical setting. Confocal laser scanning tomography, laser scanning polarimetry and optical coherence tomography (OCT) are the most widely used imaging devices in this respect. The integrity of RNFL capillary networks is less commonly used as a measure of axonal health in RGC diseases. However, as there is some evidence [7,8,11–13] to suggest that vasculogenic elements may be relevant to the pathogenesis of RGC axonal diseases the ability to quantify information about RPC networks may be important in determining disease progression in conditions such as glaucoma. Fluorescein angiography is broadly used to image the retinal vasculature however we have previously shown that only 30% of these images are of high enough quality for clear visualization of capillaries [14–16]. The presence of choroidal fluorescence restricted the ability to delineate capillary detail. Adaptive optics imaging is capable of providing excellent visualization of retinal capillary networks [17,18], though its role in RNFL disease management has to be further investigated. Recently, a combination of adaptive optics with fluorescein angiogram has produced rather impressive images of the macular vasculature [19]. However, the study of specific retinal vascular layer is yet to be seen. With both techniques, the administration of fluorescein carries a risk of anaphylactic shock [20].

Speckle variance OCT is a non-invasive device that uses the change in speckle pattern, due to red blood cell movement, and the corresponding intensity variance of structural images to identify the retinal microvasculature. It is therefore capable of providing angiographic information about the human retina without the administration of a contrast agent. Morphometric information regarding the RNFL thickness can also be acquired concurrently from the regular OCT data. This report utilizes a prototype svOCT to investigate the quantitative and morphological information that can be acquired about RPCs from normal human subjects. To assess the validity of quantitative measurements acquired from this device, svOCT data are correlated to RPC human histological data. Results from this study may have relevance for understanding axonal-vascular relationships that are important in retinal physiology and homeostasis. It may also identify a useful modality for studying RGC axonal disease from a clinical and research standpoint.

## Methods

### Ethics Statement

This study was approved by the human research ethics committees at The University of Western Australia and The University of British Columbia. All live-patient imaging was performed at the Eye Care Centre in Vancouver. All human tissue was handled according to the tenets of the Declaration of Helsinki.

### Speckle Variance OCT Imaging of Human Subjects

Sixteen retinas from nine healthy volunteers, aged between 28 and 60, were imaged using svOCT. Speckle variance OCT images of human subjects were acquired from a Graphics-Processing-Unit (GPU)-accelerated svOCT clinical prototype [21,22]. The details of the acquisition system have previously been published [22]. The OCT system was based on a 1060 nm swept source with 100 kHz A-scan rate (Axsun Inc) and 500 MSPS digitizer (AlazarTech Inc). The light source spectrum had a full-width half-maximum (FWHM) bandwidth of 61.5 nm, which corresponded to a coherence length of ~6 μm in tissue. The sample arm optics were configured to deliver a beam of ~1.5 mm diameter at subject’s pupil, with the fast axis of galvanometer mounted mirrors (6210H, Cambridge) oriented for a vertical scan. The size of the focal waist on the retina was estimated using the Gullstrand-LeGrand model of the human eye [23,24] to be $\omega_{o}\;=\;$~7.3 μm (calculated using Gaussian optics) corresponding to a lateral FWHM of ~8.6 μm (calculated as FWHM = $\sqrt{\left(2ln2\right)}\omega_{o}$).

For each research volunteer, six peripapillary regions within 1 mm from the optic disc edge in the superior, supero-temporal, temporal, infero-temporal, inferior and nasal sector were scanned with the svOCT. The scan area was sampled in a 300x300(x3) grid with a ~1x1 mm field of view in 3.15 seconds. Patient alignment was performed using a wider field, high speed, low resolution *en face* imaging mode with the OCT system, providing visual targets to guide the subject’s fixation. These intensity-only OCT intensity images were not saved. The larger vessels provided landmarks for the acquisition of the ~1x1 mm^2^ svOCT data at this location. In order to have the sample suitable for accurate capillary analysis, the svOCT data was saved in a region avoiding large blood vessels. For the speckle variance calculation, three repeat acquisitions at each B-scan location were acquired. *En face* visualization of the retinal microvasculature was processed and displayed in real-time using our open source svOCT code program developed for GPU [21]. The GPU software permitted dynamic selection of the retinal layers used for generating the *en face* svOCT image. Real-time processing to improve the svOCT image quality included: brightness and contrast adjustment to threshold out low values of speckle variance, and filtering to remove streak artifacts [21]. The *in vivo* scan dimensions on the retina were calculated using a reduced eye model (single refractive surface) [25], adjusted for the eye length of each participant measured using the Zeiss IOL Master 500. The scan dimensions on the retina were calculated as the length of an arc traced out by the OCT beam as it was scanned in angle (corrected for Snell’s law refraction) assuming a circle of radius equal to the subject’s eye length. The index of refraction for the eye media (vitreous) was approximated as n = ~1.33. The svOCT *en face* images were cropped to an area of 636.5 μm^2^ to match the dimensions of the images used for the *ex vivo* confocal microscopy analysis.

#### Retinal nerve fibre layer thickness measurement on svOCT images

The boundaries for the RNFL were determined manually on the OCT intensity B-scans, defined as the top boundary of the ILM and the top boundary of GCL. The segmentation was performed semiautomatically on the intensity data to generate the *en face* view for the vasculature in the retinal nerve fibre layer in the svOCT processed volume, and also to measure the thickness of the RNFL in the same dataset. The ILM and RPE/BM complex were segmented using a Graph Cuts algorithm implemented in MATLAB [26]. The RNFL/GCL boundary was delineated manually by adding an offset to the line generated by the automatic segmentation of the RPE/BM, shifting it anteriorly. The RNFL thickness measurement was reported as the average thickness across 10 B-scans selected from the 1x1 mm volume.

#### Confocal images from human donor eyes

The retinal microvasculature of 9 retinas from 9 donors aged between 15 and 75 were labeled using perfusion-based antibody techniques. There was no record of ocular disease in any of the donors’ medical files. The perfusion labeling technique was as described in our previous publications [27,28] and involved sequential perfusion of buffer washes, fixatives, vessel specific probes and further washes. Vessel wall specific probes including anti-Claudin-5 (Sigma SAB4502981), lectins TRITC (Sigma L5266) and anti-VE-cadherin (Santa Cruz sc—6458) were perfused through the microvasculature to label the retinal vascular endothelium. The labeled retinas were then flat mounted using glycerol and imaged using the confocal scanning laser microscope. Confocal images were collected using four lasers (408, 488, 561 and 635 nm wavelengths) in a Nikon C1 confocal system, coupled with the Nikon i90 motorized microscope fitted with plan apochromatic lenses.

**Fig 1:**
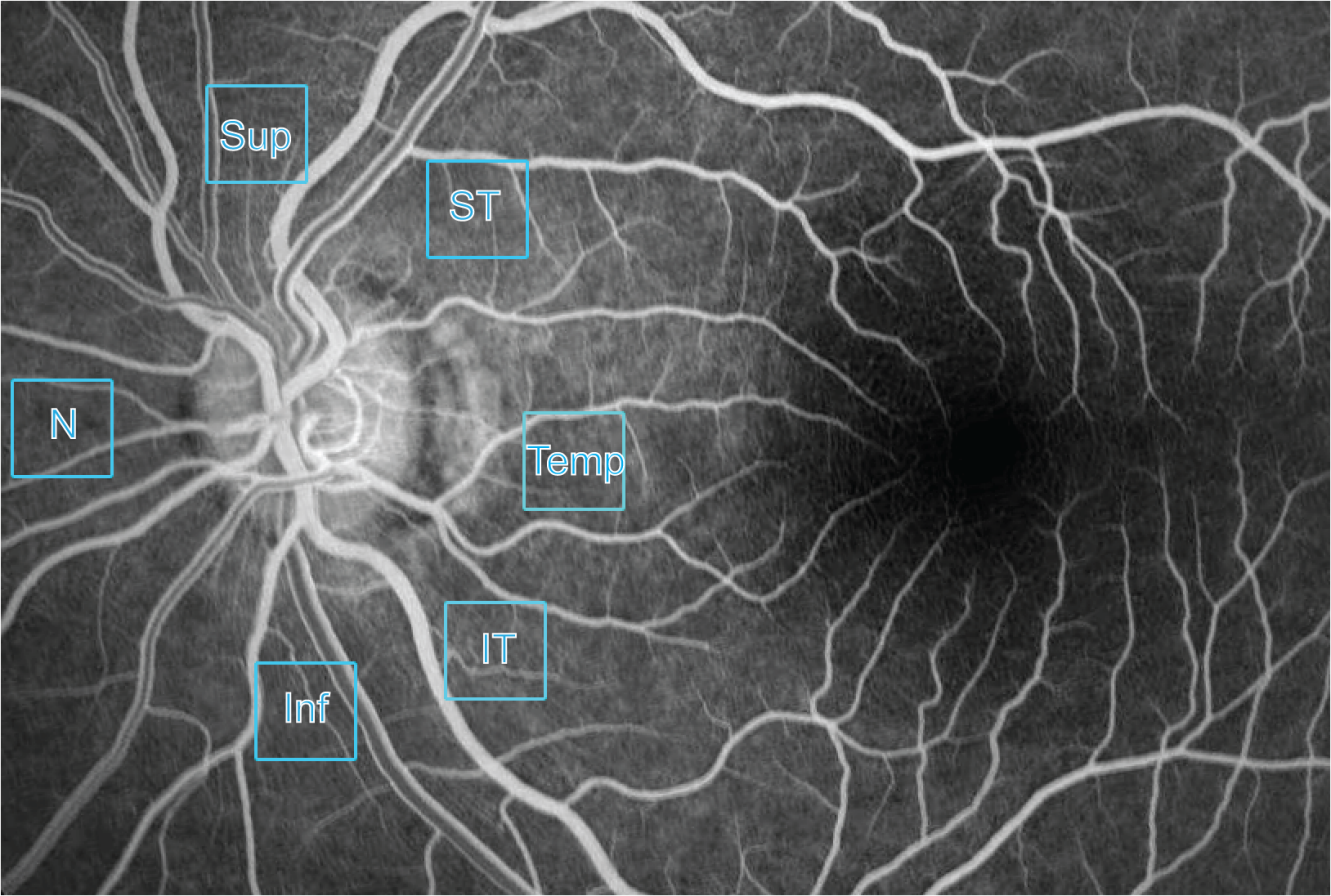
Regions where radial peripapillary capillary network morphology and topography were studied quantitatively. Insets placed on a fluorescein angiogram illustrate the six different eccentricities that were studied with speckle variance optical coherence tomography and histology. Sup = Superior; ST = Supero-temporal; Temp = Temporal; IT = Infero-temporal; Inf = Inferior and N = Nasal regions.

Confocal image stacks were obtained from the superior, supero-temporal, temporal, infero-temporal, inferior and nasal regions (Fig 1) using the x20 plan apochromatic objective lens, with the field of view centered on the 500μm mark from the optic disc edge. Images were collected at 0.35 μm step size, with the total thickness of the nerve fiber layer ranging between 5 to 30 microns. During the collection of confocal images, care was taken to avoid inclusion of the large retinal vessels. The field of view measured an area of 636.5 x 636.5 square microns. Optical slices encompassing the RNFL were projected to provide a two dimensional image of the radial peripapillary capillaries.

### Image processing and analysis

**Fig 2:**
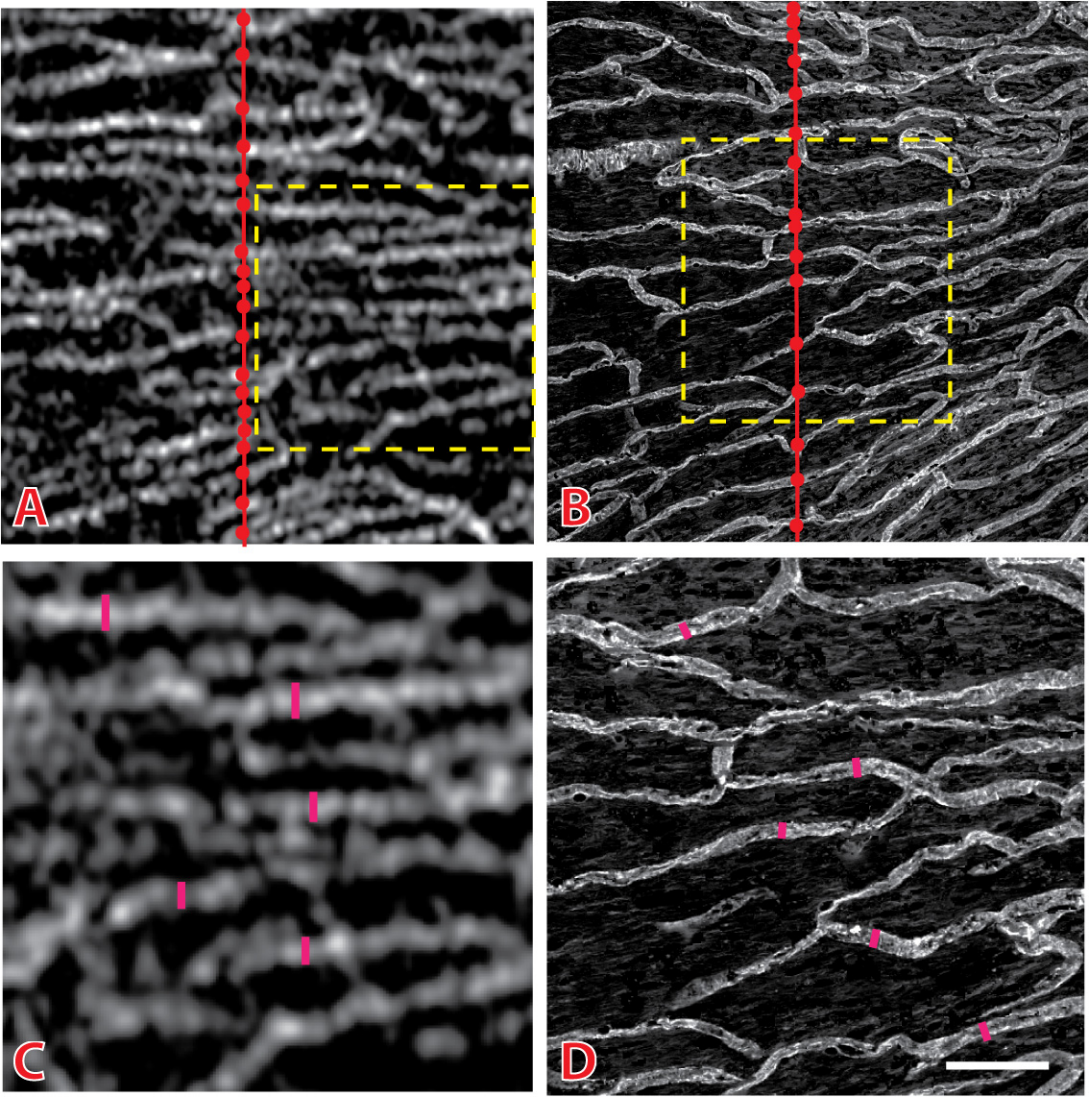
Methodology for quantitative measurements. Representative speckle variance optical coherence tomography (svOCT) image (A) and histology image (B) from the nasal peripapillary region illustrates the technique used to acquire quantitative measurements. Capillary density was determined by calculating the number of intersections between capillaries (circles) and a perpendicular, straight line (red line) drawn through the capillary network. Results were expressed as intersections per 100 μm. Capillary diameter was determined by calculating the perpendicular distance across the maximum chord axis of each vessel. This is as illustrated in the corresponding magnified regions (outlined in yellow dashed lines) of svOCT (C) and histology (D) images. Scale bar = 120μm (A & B) and 60μm (C and D)

**Fig 3:**
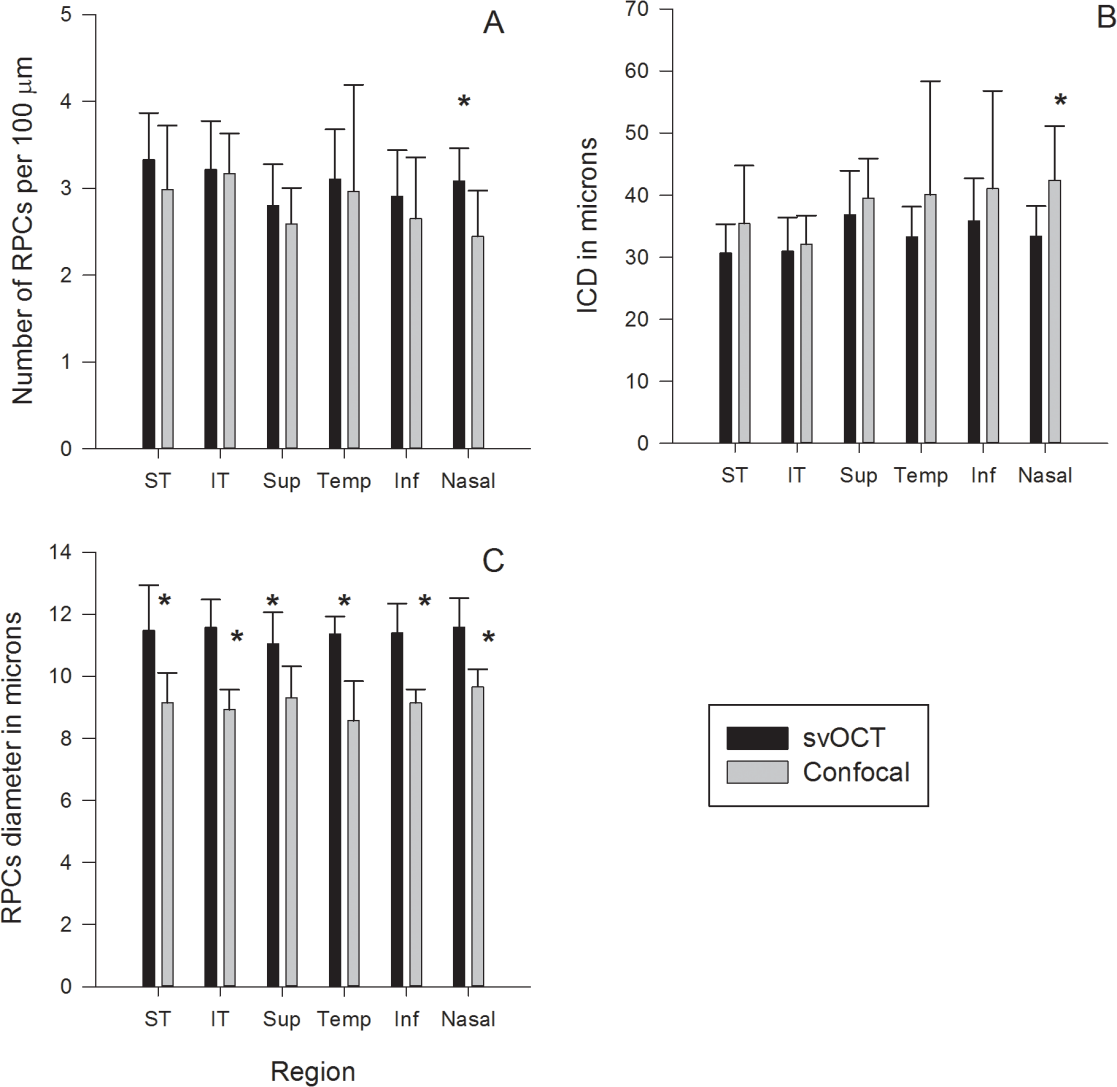
A paired graphical representation of quantitative comparisons between speckle variance optical coherence tomography (svOCT) and histology data for the six eccentricities. The averaged value is presented as solid bars with standard deviations presented by error bars. Black solid bars represent data from svOCT images whilst grey solid bars represented data from confocal images. Data presented include (A) number of RPCs per 100 μm, (B) Inter-capillary distance or ICD in microns, and (C) RPCs diameter in microns. The data are presented as per study region and include the supero-temporal (ST), infero-temporal (IT), superior (Sup), temporal (Temp), inferior (Inf) and nasal (Nasal). Statistically significant difference (p<0.05) between the two techniques are marked with an asterisk.

Image pro plus (v7.0) was used to analyze all images. The following quantitative measurements were acquired (Fig 2):

- Capillary density–Density was measured using two indices: The first index calculated the number of RPCs per 100 μm. This was done by drawing a line perpendicular to the orientation of the RPCs and counting the number of intersecting RPCs. To minimize variation in density calculation due to the radial divergence of RPCs, the position of the line is drawn as close to the center of each image as possible. The number of vessels were then averaged and expressed per 100 μm length (Fig 3). The second index of capillary density calculated the inter-capillary distance (ICD). This was determined by dividing the total distance measured by the number of RPCs crossing it (Fig 3).
- Capillary diameter—Defined as the perpendicular distance across the maximum chord axis of each vessel (Fig 2).
- Correlation between retinal nerve fibre layer thickness and capillary density–This measurement was only determined using svOCT images. A regression analysis was used to calculate the correlation between capillary density (using ICD measurements described above) and retinal nerve fibre layer thickness for each peripapillary eccentricity. Retina nerve fibre layer thickness measurements for each eccentricity was determined by performing manual measurements of this layer on the B scan images.

### Statistical analysis

All statistical testing was performed using statistical package R (R Foundation for Statistical Computing, Vienna, Austria) and SigmaStat (Systat Software, Inc, Windows Version 3.5). A linear mixed model using random factors right or left nested within patient identity was used to account for correlation between multiple measurements from the 2 eyes of subjects. The model analysed capillary density, ICD and capillary diameter as response variables, using technique (svOCT or Confocal) as the explanatory factor, with or without region as additional explanatory factor. This is analogous to one or two way ANOVA with correlation for multiple measurements from the same eye and two eyes of same subjects. To isolate any variation that could be introduced by regional variation, especially between the arcuate fibre regions (supero-temporal and infero-temporal) and the other four quadrants, the capillary density and ICD for each region were analysed separately by subsetting the data. We also tested a possible relationship between ICD and RNFL thickness with a similar linear mixed model using ICD as response variable and RNFL thickness and regions as the explanatory variables. All data are expressed as mean ± standard deviation. T-tests was also carried out to determine if the ages of subjects and donors were comparable. ICD data were log transformed to attain normality prior to analysis. Where the *P*-value was less than 0.050, the difference was considered statistically significant.

## Results

### Demographics

The average age was 42.7 ± 4.7 years for the 9 live subjects and 40.9 ± 7.6 years for the 9 donors. The age range was comparable between the two groups (*P* = 0.836).

### Morphology and pattern of RPC networks on svOCT and confocal microscopy images

The contour of capillary lumens was different between svOCT and histology images. In histological images, the capillary walls were smooth and undulating, whereas the appearance of the capillary thickness in the svOCT images in all networks demonstrated non-uniform varicosities. In comparison to confocal images, there was more noise in the svOCT images that resulted in less contrast between capillaries and background.

**Fig 4:**
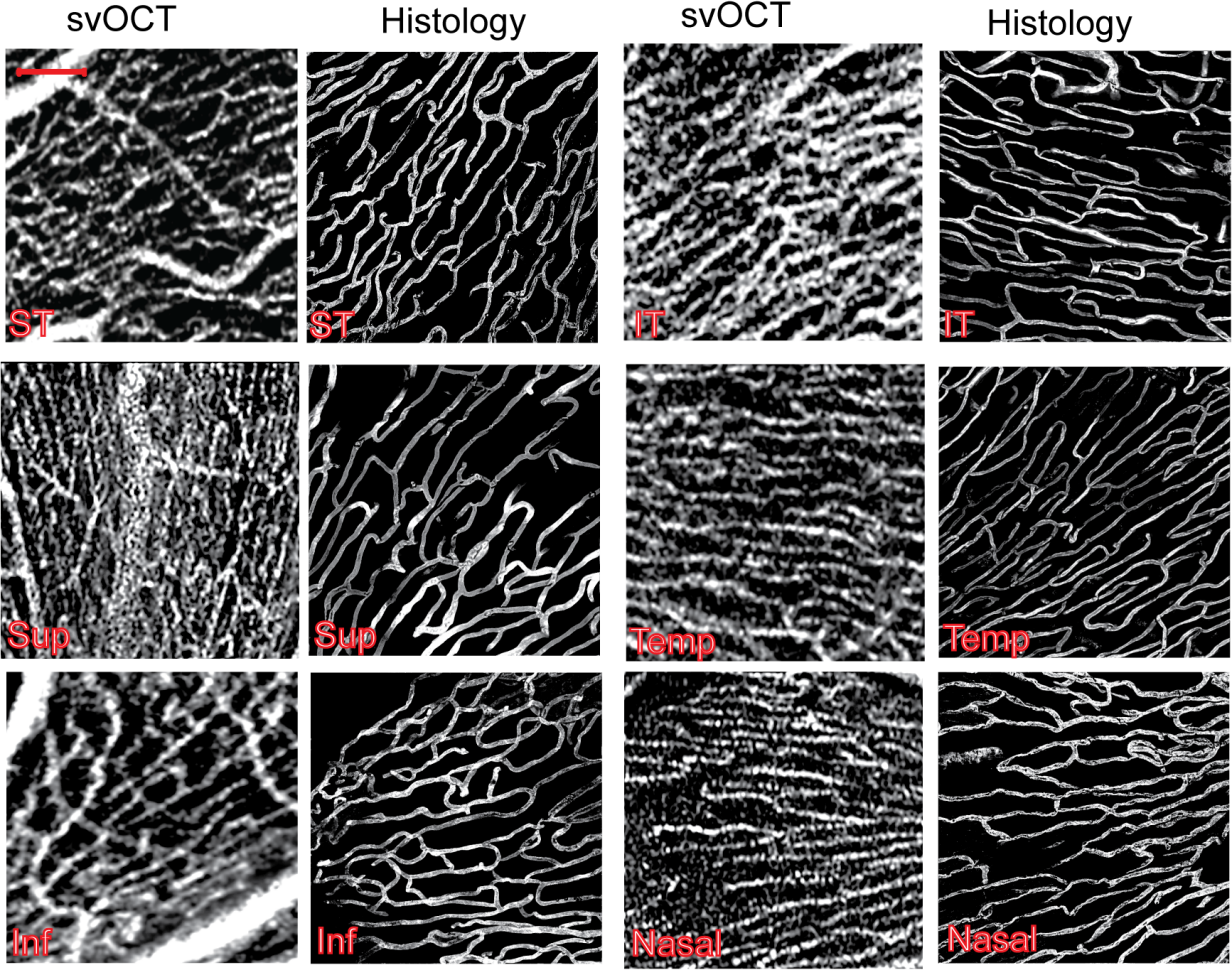
Comparison of radial peripapillary capillary (RPC) network morphology between speckle variance optical coherence tomography and histology images. Representative images from the six study regions obtained using svOCT and the confocal scanning laser microscope are provided. Sup = Superior; ST = Supero-temporal; Temp = Temporal; IT = Infero-temporal; Inf = Inferior and N = Nasal. Scale bar = 100 μm.

We observed that the pattern of RPC capillary networks in svOCT images were similar to histology. Representative images from the six study regions obtained using both techniques are presented in Fig 4. A movie depicting the organization of radial peripapillary capillaries at various depths in the retinal nerve fibre layer is also provided using svOCT (S1 Video) and histology (S2 Video) image stacks. With both modalities, RPCs were seen to radiate outwards from the optic disk to the periphery. Within each eccentricity, RPCs demonstrated a similar trajectory with most capillaries running parallel to one another. The parallel orientation of capillaries was particularly evident in the temporal eccentricity. Capillary density appeared to be greatest in the ST and IT regions. The configuration of capillary hairpin loops in these three regions seemed narrower.

### Capillary density comparisons

Whilst the number of RPCs per 100 microns appeared to be higher in the svOCT images, RPC density measurements were not significantly different between svOCT and confocal microscopy images for all regions (all *P* > 0.213) except for the nasal region (*P* = 0.015, Fig 3). In the nasal region the capillary density was significantly greater in svOCT images. svOCT showed the RPCs density to be significantly greater in the arcuate fibre (pooled data from infero-temporal and superotemporal) regions compared to the pooled data from the other four regions (*P*_svOCT_ = 0.003, Fig 3). A similar trend was observed in the histology data, although slightly short of being statistically significant, *P*_Confocal_ = 0.059.

Analysis of confocal microscopy data alone demonstrated no significant difference in capillary density (i.e. number of capillary per 100 microns) measurements between the 6 regions (all *P* > 0.060).

Analysis of svOCT data alone revealed that number of RPCs per 100 microns in the ST region was significantly higher than the superior (*P* = 0.003) and inferior (*P* = 0.008) regions. The RPC density in the IT region was also significantly greater than the superior region (*P* = 0.028). Comparisons between remaining regions did not reveal a significant difference (all *P* > 0.050).

The log transformed ICD measurements were also found to be comparable between histology and svOCT images for all regions (*P* > 0.066) except for the nasal region (*P* = 0.026). In the nasal region, measurements were consistently lower in svOCT images indicating a denser distribution which is consistent with the capillary density measurement. Both techniques showed intercapillary distance in the range of 30 to 42 μm, with the arcuate (ST and IT) regions showing consistently smaller intercapillary distance (P_svOCT_ < 0.001, P_Confocal_ = 0.039). The svOCT data showed a significantly smaller ICD in the two arcuate regions when compared to the other four regions (*P* = 0.0004). In particular, the ST region was found to have significantly smaller intercapillary distance when compared with the inferior (*P* = 0.005) and superior (*P* = 0.003) regions, whilst the IT region had a significantly smaller ICD than the superior region (*P* = 0.005). The confocal images also identified a significantly smaller ICD in the arcuate regions when compared to other regions (*P* = 0.037).

### RPC diameter comparisons

RPC diameter was significantly greater in svOCT images than confocal images for all 6 regions (all *P* < 0.004, Fig 3). Analysis of RPC diameter from confocal images found temporal RPCs to be significantly smaller than all other regions (all *P* < 0.050) except for IT region (*P* = 0.186). Analysis of RPC diameter from svOCT images did not identify significant differences between the 6 regions (*P*_svOCT_ = 0.730). Age was not found to have an effect on RPC diameter when imaged using either technique (*P*_confocal_ = 0.110, *P*_svOCT_ = 0.801).

#### Correlation between RNFL thickness and ICD

**Fig 5:**
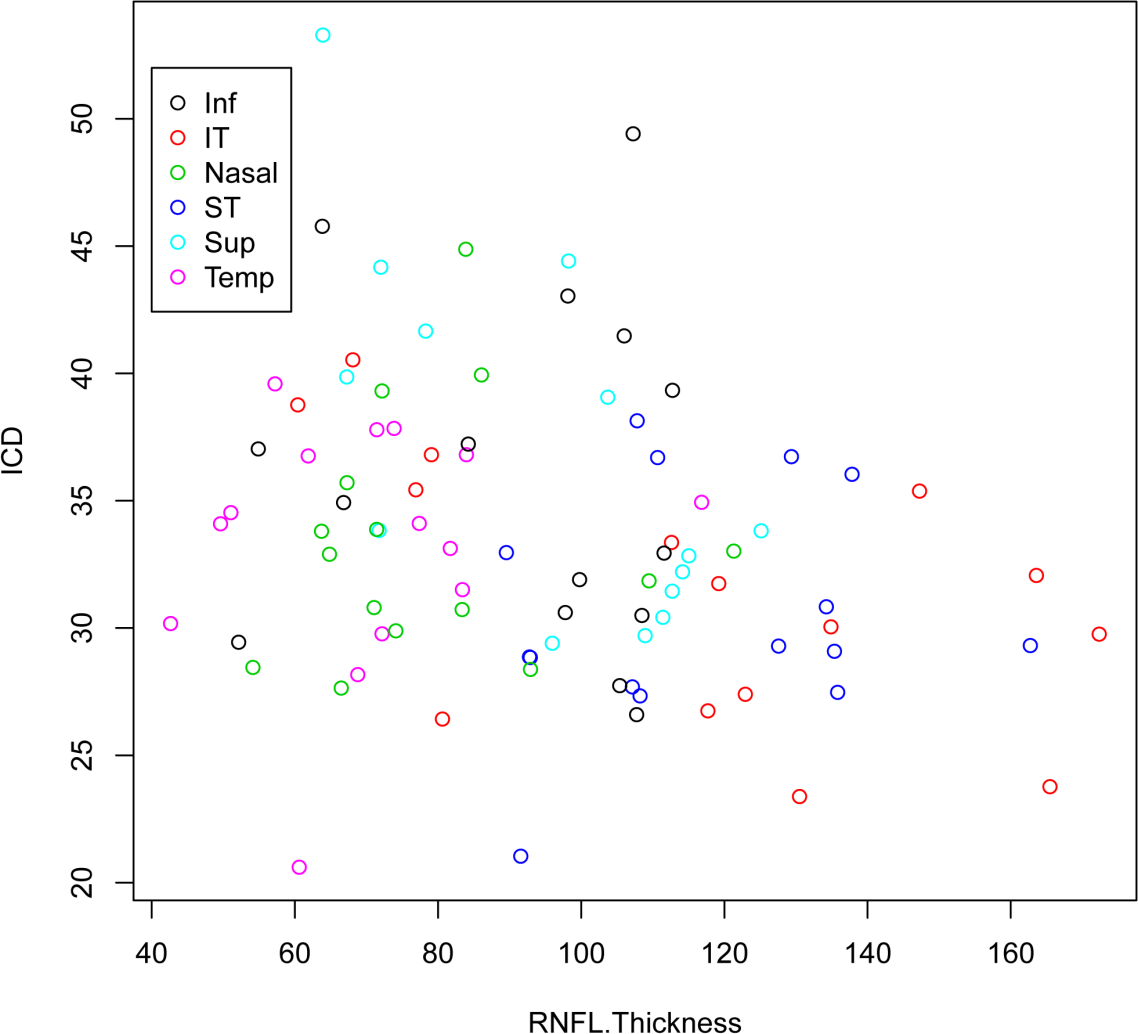
Scatter plot of intercapillary distance (ICD) and retinal nerve fibre layer thickness (RNFL) with the speckle variance optical coherence tomography. Each region is colour coded. Sup = Superior; ST = Supero-temporal; Temp = Temporal; IT = Infero-temporal; Inf = Inferior and N = Nasal. Unit of measurement in both axes is in microns.

ICD was found to be inversely associated with RNFL thickness (*P* = 0.006, slope -0.065) using svOCT images after accounting for region in the linear mixed model (Fig 5). There were only 15 measurements from each region with most being from the two eyes of subjects which limited the power of sub-regional analysis. Only in the superior region was a significant relationship found (*P* = 0.011).

## Discussion

The major findings in this study are as follows: (1) The topographical and morphological characteristics of RPC networks on svOCT images are comparable to histological images; (2) With the exception of the nasal peripapillary region, there are no significant differences in RPC density measurements between svOCT and histological images; (3) Capillary diameter measurements are greater in svOCT images compared to histology.

RPCs are a unique organization of capillary networks that play a vital role in supporting RGC axonal metabolic activity [4,5]. There is strong experimental evidence to suggest that insults to the RPC network can adversely affect RGC axonal structure and function. The pattern of RNFL defect in conditions such as cotton wool spots [9], intraretinal hemorrhage and ischemic optic neuropathy bear important correlation to the distribution of RPCs [11,12,14,15]. The role of RPC dysfunction in glaucoma pathogenesis however remains controversial [8,10,12,13].

The ability to visualize the RPC network *in vivo* may provide a useful means for studying and improving our understanding about pathogenic mechanisms involved in RGC axonopathies. Fluorescein angiography has widespread clinical use for the management of retinal vascular disease, however, the anatomy of the RPC network can be difficult to discern on fluorescein angiography with the fine details of the vasculature being commonly obscured by the choroidal flush-phase of the angiogram [29]. Scoles et al. [17] performed an elegant study that imaged the macaque RPC network using adaptive optics techniques. However, in this study, intravenous fluorescein was administered to improve the contrast of vasculature within regions of interest. Thus, the adverse and potentially life-threatening risks of intravenous fluorescein administration were not circumvented. More recently, Chui et al. [30] were able to image the retinal microvasculature using an adaptive optics scanning laser ophthalmoscope (AOSLO) by varying the size and centration of the confocal aperture.

The prototype svOCT device used in this report provided morphological information about RPC networks that resembled what has been reported histologically. Histology in this study was in the form of confocal scanning laser microscope images that were acquired from cadaveric human tissue that underwent perfusion-based labeling of the retinal capillary endothelium. We have previously shown that this technique allows detailed labeling of the retinal microcirculation [6,27,28,31] and is therefore a particularly advantageous histological correlate against which *in vivo* imaging modalities can be compared. On svOCT images, radial peripapillary capillaries demonstrated a long, linear trajectory. RPCs were also seen to radiate anteriorly from the optic disk forming only a few anastomoses with adjacent vessels. In all regions studied with svOCT, RPCs were oriented parallel to adjacent capillaries however this configuration was most profound in the temporal region. Our quantitative comparisons revealed no differences in capillary density measurements between svOCT and histology for all regions other than the nasal eccentricity. Collectively, these findings suggest that svOCT is a reliable modality for analyzing RPC networks in the human retina.

Interestingly, capillary diameter measurements acquired from svOCT images were consistently greater than histology in all regions that were studied. The lower lateral resolution of the svOCT compared to histology may account for some of the differences in the diameter measurements. The influence of hemodynamic parameters such as blood pressure, interstitial tissue pressure and the effects of localized pericyte-mediated contraction may also have accounted for the greater RPC diameter measurements in svOCT images [32]. In addition, fixation and perfusion pressure are other possible factors in the perfusion technique that could contribute to the smaller capillary diameter.

We also observed greater variation in pixel intensity along the length of the capillary wall in svOCT images compared to histology. The appearance of capillary lumens was different between svOCT and histology images. In histological images, the capillary walls were smooth and relatively even, whereas the appearance of the capillary appearance in the svOCT images in all networks demonstrated non-uniform varicosities [33]. Speckle variance OCT has the capacity to provide semi-quantitative information about retinal capillary blood flow. The distribution of erythrocytes along a column of blood flow is known to be non-uniform and the variation in pixel intensity in svOCT images is likely to reflect the differential distribution of erythrocytes within that capillary bed [34,35]. The width and intensity of a capillary in the svOCT image are also sensitive to the moving of the blood cells at the particular vessel location being imaged within the time length of the three B-scans used to calculate sv (~0.01 second). We speculate that the irregular or beaded appearance of capillaries in svOCT images reflect pulsatile flow and the non-uniform distribution of red blood cells and leukocytes along the capillary length. Previous modeling studies have shown that red blood cells within a capillary bed travel in groups and that the velocities of red blood cells are not constant [36]. It may also reflect regional pericyte constrictions within the length of a capillary segment. [32] A closer study of the distribution of the capillary varicosities on svOCT images may aid our understanding of mechanisms that control oxygen and nutrient delivery in the human retina.

The anastomoses of capillaries that run perpendicular to the radially-arrayed capillaries appeared smaller than the radial capillaries only in the svOCT images. It is possible that lower flow in the anastomoses may account for their narrower appearance relative to the radially arranged capillaries in the flow contrast svOCT images. We will be investigating and describing this issue in more detail in future studies that are currently under way.

A smaller variance in RPC diameter was noted from the svOCT images. This is likely a function of the image averaging as well as the lower resolution of the svOCT technique as the scanning of imaging beam across a capillary can be modelled as a convolution of the spot size with the capillary width. Pixels corresponding to locations where the imaging beam only partially overlapped with the vessel had low speckle variance values and were thresholded out and did not contribute to the final svOCT image data that was used for analysis. The averaged capillary diameter measurements reported this manuscript is between 6.7 and 6.9 pixels. The comparatively lower resolution of the svOCT images with respect to the confocal microscopy of the ex vivo tissue samples may be a contributing factor to the lower variation in the measurements with the in vivo imaging technique.

The pathophysiology of glaucomatous optic neuropathy is complex. In particular, the role of vasculogenic mechanisms in RGC axonal degeneration remains unclarified. Recently, a number of detailed studies have employed Fourier domain-OCT Doppler flow techniques to study the relationship between retinal blood flow disturbances and glaucomatous field loss [37,38]. These studies have demonstrated a reduction in blood flow in both glaucomatous and normal hemispheres in glaucoma patients. It will be important to determine if quantitative changes in the density and morphology of RPC networks are related to retinal blood flow changes in glaucoma. In the present study, we identified a positive correlation between RNFL thickness and RPC intercapillary distances, reflecting the importance of neurovascular co-patterning and functional cross talk mechanisms in retinal homeostasis. Using svOCT to study longitudinal changes in RPC networks and their relationship to RNFL thickness in glaucoma may potentially provide a microanatomic basis for understanding previously reported circulatory disturbances. Such studies may also help resolve cause-consequence relationships in RGC axonal diseases.

## Conclusions

In svOCT images, capillary segments were represented by focal points of increased pixel intensity that were separated by areas of significantly decreased pixel intensity. Capillary segments were therefore characterized by the appearance of bulbous varicosities due to the variations in pixel intensity along its length.

This study illustrates the utility of svOCT, a safe and non-invasive device, for quantitatively imaging human RPC networks. Information acquired with this modality may provide useful clinical information that may be used in the management of RGC axonal and optic nerve head diseases. Further work is required to validate the role of this device in the management of different disease entities.

## Supporting Information

S1 Video A short movie depicting the organization of radial peripapillary capillaries in the retinal nerve fibre layer of subject 3 right eye in the nasal region. The movie is compiled from seven svOCT images taken at sequentially increasing depth starting from the vitreal surface of the retina. The images have been cropped to an area of 636.5 x 636.5 microns. Continuous white lines running in parallel orientation and horizontally across frames 4 to 7 depict signals generated from the speckle variance from the red cells movement within the radial peripapillary capillaries. A large white band may be seen in frames 6 and 7 and depicts a major retinal vessel in the deeper level of this field in the ganglion cell layer.
(AVI)

S2 Video A short movie generated from a confocal stack taken from the superior region of one of the histology specimens. The movie depicts the arrangement of the radial peripapillary capillaries through the depth of the retinal nerve fibre layer. The blood vessels were labelled intravascularly by perfusion and appeared red. The nuclei have been counterstained and are pseudo-coloured green in this sequence. The RPCs appeared fairly straight and ran in a parallel orientation to each other through the nerve fibre layer. Each frame is a confocal image collected using a x20 objective lens and measures 636.5 x 636.5 microns.
(AVI)
